# Emergence of Carbapenem-resistant Clinical Isolates of *Providencia* Species

**DOI:** 10.14789/jmj.JMJ21-0057-R

**Published:** 2022-06-09

**Authors:** SHU IWATA, TATSUYA TADA, SATOSHI OSHIRO, TOMOMI HISHINUMA, MARI TOHYA, TERUO KIRIKAE

**Affiliations:** 1 Department of Microbiology, Juntendo University School of Medicine, Tokyo, Japan; 1 Department of Microbiology, Juntendo University School of Medicine, Tokyo, Japan

**Keywords:** *Providencia rettgeri*, *Providencia stuartti*, metallo-β-lactamase

## Abstract

*Providencia* is a genus of Gram-negative and non-spore forming bacteria belonging to the family *Morganellaceae*, which causes opportunistic infections in humans. Of the 10 *Providencia* species identified to date, three, *P. alcalifaciens*, *P. rettgeri* and *P. stuartii*, are clinically important. *P. alcalifaciens* causes diarrhea, including outbreaks arising from food-borne infections, and *P. stuartii* and *P. rettgeri* have been found to cause hospital acquired urinary tract infections. Four isolates of *P. rettgeri* and one isolate of *P. stuartii* were obtained from urine samples of five patients in Japan in 2018. All five isolates were highly resistant to carbapenems. Three isolates harbored *bla*_IMP-70_, encoding a variant of IMP-1 metallo-β-lactamase, with two amino acid substitutions (Val67Phe and Phe87Val), one isolate harbored two copies of *bla*_IMP-1_ and one isolate harbored *bla*_IMP-11_. Expression of *bla*_IMP-70_ conferred carbapenem resistance in *Escherichia coli*. Recombinant IMP-10, an IMP-1 variant with Val67Phe but without Phe87Val, had significant higher hydrolytic activities against meropenem than recombinant IMP-1, indicating that the Val67Phe amino acid substitution alters activities against meropenem in IMP-70. These results suggest that *Providencia* species. become more highly resistant to carbapenems by acquisition of two copies of *bla*_IMP-1_ or by mutations in *bla*_IMP_ that result in amino acid substitutions, such as *bla*_IMP-70_.

## Taxonomy of the *Providencia* genus

**Figure 1:**
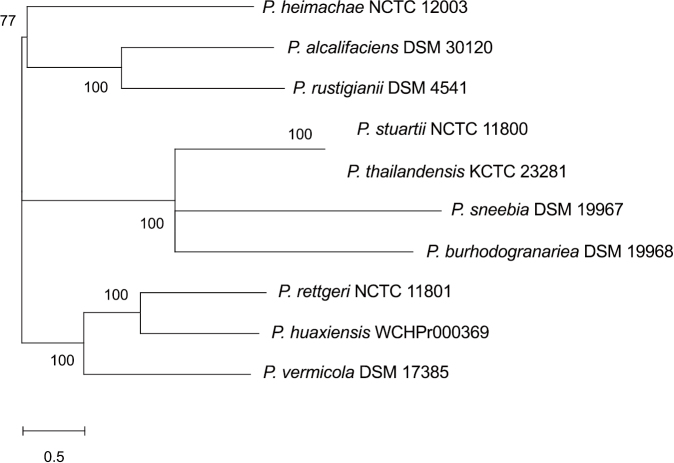
Maximum-likelihood (ML) tree based on single nucleotide polymorphisms (SNPs) in the core genome among contigs of strains, showing the relationships among type strains of the genus *Providencia*. Bootstrap values, expressed as percentages of 1,000 replications, are shown at the branching points when >50 %.

*Providencia*, a genus of Gram-negative and non- spore forming bacteria, was originally assigned to the family *Enterobacteriaceae*, but has recently been assigned to the family *Morganellaceae*^1)^. Species of *Providencia* genus have been isolated from many vertebrate and invertebrate animals, including humans and insects^2-4)^, and causes opportunistic infections in humans^5)^. To date, 10 species belonging to the genus *Providencia* have been identified: *P. alcalifaciens*, *P. burhodogranariea*, *P. heimbachae*, *P. huaxiensis*, *P. rettgeri*, *P. rustigianii*, *P. sneebia*, *P. stuartii*, *P. thailandensis* and *P. vermicola*. Of these 10 species of *Providencia*, five, *P. alcalifaciens*, *P. friedericiana* (synonym of *P. rustigianii*), *P. rettgeri*, *P. stuartii* and *P. vermicola*, were isolated from humans, with three of these, *P. alcalifaciens*, *P. rettgeri* and *P. stuartii*, likely to be clinically important^5, 6)^. A phylogenetic tree based on whole genome sequences of the 10 *Providencia* species revealed that these species consist of three clusters, with the five species isolated from humans, being spread among these three clusters (Figure 1). These findings indicate that the genus *Providencia* does not have a subgenus associated with human infections. The five species were found to have specific genes associated with human infections, such as genes encoding adherence and invasion factors.

## *Providencia* species as human pathogens

A study of the enteropathogenicity of *P. alcalifaciens* isolated from a child and two adults with diarrhea demonstrated that this species causes diarrhea in humans by invading the intestinal mucosal epithelium^7)^. *P. alcalifaciens* was subsequently isolated from 2.1% of the stool specimens of diarrheal children younger than 5 years of age, indicating that this organism is significantly associated with diarrhea^8)^. A large outbreak of food-borne infection caused by *P. alcalifaciens* occurred among children and teachers at two kindergartens and one high school in November 1996 in Fukui, Japan^9)^. Specifically, of the 610 children and teachers who ate lunch cooked at a single catering facility, 270 showed symptoms of gastroenteritis^9)^. Recent outbreaks of *P. alcalifaciens* have indicated that infection with this organism is a public health concern in both developing and developed countries^10)^. Although epidemiological studies suggest that *P. alcalifaciens* causes diarrhea by invading the intestinal mucosa^10)^, the pathogenesis of *P. alcalifaciens* has not been established at the molecular level.

*P. stuartii* and *P. rettgeri* have been found to cause hospital acquired urinary tract infections^11)^ and have been shown to be the most common causes of urinary tract infections in hospitalized patients. In addition, *P. stuartii* and *P. rettgeri* have been found to cause pneumonia, meningitis, endocarditis, wound infections and bloodstream infections^11)^, and *P. stuartii* was found to cause invasive endocarditis^12)^ and neonatal sepsis^13)^. *P. alcalifaciens*, *P. rettgeri* and *P. stuartii* were isolated from 17.6% of stool samples of patients with diarrhea at the Kansai airport quarantine station in 2002, with vomiting being especially frequent in patients infected with *P. rettgeri*, indicating that these three *Providencia* species cause travelers' diarrhea^5)^.

## Emergence of carbapenem-resistant *Providencia* species

The emergence and spread of carbapenem-resistant Gram-negative pathogens have become serious public health problems worldwide^14)^. Most of these carbapenem-resistant isolates produce metallo-β-lactamases (MBLs), including IMP-, NDM- and VIM-type MBLs^14)^, which confer high resistance against all β-lactams (penicillins, cephalosporines and carbapenems) except for monobactams^15)^. Clinical isolates of carbapenem-resistant *P. rettgeri* producing IMP-1 MBL were first identified by laboratory-based surveillance in the Kinki region of Japan in 2000^16)^. Clinical isolates of *P. stuartii* producing VIM-19 MBL were first identified in 2008 in Algeria^17)^. To date, there have been 16 reports of carbapenem-resistant *P. rettgeri*, eight of carbapenem-resistant *P. stuartii* and one of carbapenem-resistant *P. vermicola* (Table 1). Most of these were clinical isolates, but one was obtained from a hospital environment and one from pet turtles (Table 1).

All of these isolates produced MBLs, with the majority of carbapenem-resistant *P. rettgeri* isolates producing IMP-type or NDM-type MBLs (Table 1). IMP-type MBLs were detected in isolates from Japan, Korea, and the United States, whereas NDM- type MBLs were detected in isolates worldwide. We obtained four clinical isolates of carbapenem- resistant *P. rettgeri*, which produced IMP-1, IMP-11 or IMP-70. One IMP-1 producing isolate was from Saitama, Japan, one IMP-11 producing isolate was from Kochi, Japan, and two IMP-70 producing isolates were from Osaka, Japan^18)^.

Most of the carbapenem-resistant *P. stuartii* isolates, obtained in Algeria, Greece and Korea, produced VIM-type MBLs. Carbapenem-resistant *P. stuartii* isolates producing NDM-type MBL- producing *P. stuartii* were obtained in Afghanistan and Peru, and we described an IMP-type MBL- producing *P. stuartii* from Japan^18)^. Carbapenem- resistant *P. vermicola* isolates producing NDM-1 were isolated in the Congo.

**Table 1:** Reports of *P. rettgeri*, *P. stuartii* and *P. vermicola* producing MBL^a^

Species	Metallo-β-lactamase	Location of MBL-encoding gene	Inc type	Isolation source	Isolated year	Isolated country	Reference
*P. rettgeri*	IMP-1	-	-	-	2000	Japan	^16)^
	IMP-1	plasmid	-	sputum, blood	2002	Japan	^25)^
	IMP-1	chromosome		urine	2018	Japan	^18)^
	IMP-11	plasmid (84,930-bp)	IncT	urine	2018	Japan	^18)^
	IMP-27	plasmid (10,7365-bp)	IncQ	wound	2016	USA	^26)^
	IMP-27	-	-	pet turtles	2018	Korea	^27)^
	IMP-70	plasmid (204,791-bp)	IncA/C2	urine	2018	Japan	^18)^
	NDM-1	-	-	blood, rectum, pus	2008	Israel	^28)^
					2011		
	NDM-1	plasmid	-	sputum, pus	2012	Nepal	^29)^
	NDM-1	plasmid (178kb)	-	urine	2012	Mexico	^30)^
	NDM-1	plasmid (190kb)	IncA/C	urine	2014	China	^31)^
	NDM-1	-	-	-	2017	Bulgaria	^32)^
	NDM	-	-	wound	2013	Brazil	^33)^
	NDM-1, VIM-2	plasmid	-	urine	2015	Colombia	^34)^
	NDM-18	plasmid	-	effluent from a pediatric ward	2017	South Africa	^35)^
*P. stuartii*	IMP-70	plasmid (152,754-bp)	IncA/C	urine	2018	Japan	^18)^
	NDM-1	plasmid (178277kb)	IncA/C	blood	2012	Afghanistan	^36)^
	NDM-1	plasmid (18,480-bp)	IncA/C2	urine	2020	Peru	^37)^
	VIM-1	-	-	-	2011	Greece	^38)^
	VIM-1	plasmid (180kb)	IncA/C	rectum	2012	Greece	^39)^
	VIM-1	plasmid	-	-	2013	Greece	^40)^
	VIM-2	-	-	urine	2004	Korea	^41)^
	VIM-19	plasmid (180kb)	-	-	2008	Algeria	^17)^
*P. vermicola*	NDM-1	plasmid (151,684-bp)	-	blood	2017	Congo	^42)^

^a^ Iwata S, Tada T, Hishinuma T, et al: Antimicrob Agents Chemother, 2020; 64.^18)^ A dash (–) indicates there was no information about the location of MBL-encoding genes, Inc type and isolation source.

## Carbapenem-resistant clinical isolates of *Providencia* species in Japan

We obtained four clinical isolates of *P. rettgeri* and one clinical isolate of *P. stuartii* from the urine samples of five patients in Japan in 2018^18)^. All five were multidrug-resistant, being resistant to aminoglycosides, carbapenems and fluoroquinolones^18)^. These isolates harbored genes encoding aminoglycoside modifying enzymes, including *aac*(*6’*)-*Ib4* and *aac*(*6’*)-*Iae*, and MBL genes encoding carbapenemases; including IMP-1, IMP-11 and IMP-70 (Table 2). They also had three mutations with amino acid substitutions in *GyrA* and *ParC*, which were associated with quinolone resistance (Table 2). One isolate harbored aminoglycoside- and carbapenem-resistant genes on the chromosome, whereas the other four harbored these genes on plasmids.

**Table 2:** Genetic characterization of carbapenem-resistant *Providencia* species isolates^a^

isolates	genome	size (bp)	antibiotic resistance genes			quinolone resistance genes	
			aminoglycosides	carbapenemase		*GyrA*	*ParC*
*P. rettgeri* BML2496	chromosome	4.65M	*aac*(*6’*)-*Ib4*	*bla*_IMP__-__1_		Ser83Ile Asp87Ala	Ser87Ile
*P. rettgeri* BML2526	chromosome	4.34M				Ser83Ile Asp87Glu	Ser87Ile
	plasmid	205K	*aac*(*6’*)-*Iae*	*bla*_IMP__-__70_			
*P. rettgeri* BML2531	chromosome	4.70M				Ser83Ile Asp87Glu	Ser87Ile
	plasmid	85K	*aac*(*6’*)-*Il*	*bla*_IMP__-__11_			
*P. rettgeri* BML2576	chromosome	4.35M				Ser83Ile Asp87Glu	Ser87Ile
	plasmid	205K	*aac*(*6’*)-*Iae*	*bla*_IMP__-__70_			
*P. stuartii* BML2537	chromosome	4.42M	*aac*(*2’*)-*Ia*			Ser83Ile Asp87Glu	Ser87Arg
	plasmid	153K	*aac*(*6’*)-*Iae*	*bla*_IMP__-__70_			

^a^ Iwata S, Tada T, Hishinuma T, et al: Antimicrob Agents Chemother, 2020; 64.^18)^

## Carbepenemase activities of IMP-1 MBL variants

All five *P. rettgeri* and *P. stuart*ii clinical isolates were resistant to imipenem and meropenem, and three, two *P. rettgeri* isolates and one *P. stuartii* isolate, were highly resistant to both carbapenems, with minimum inhibitory concentrations (MICs) of 512 μg/ml (Table 3). These three highly carbapenem-resistant isolates harbored *bla*_IMP__-__70_, whereas, of the other two, one harbored *bla*_IMP__-__1_ and the other harbored *bla*_IMP__-__11_. IMP-70 is a variant of IMP-1 with two amino acid substitutions, Val67Phe and Phe87Val; IMP-10 is a variant of IMP-1 with one amino acid substitution, Val67Phe; and IMP- 1(F87V) is a variant of IMP-1 with one amino acid substitution, Phe87Val. *E. coli* expressing *bla*_IMP__-__1_, *bla*_IMP__-__10_, *bla*_IMP__-__1__(__Phe87Val__)_, and *bla*_IMP__-__70_ showed significantly higher MICs for all carbapenems tested than a vector control (Table 4). The MICs for all carbapenems of the vector control ranged from ≤0.06 to 0.125. *E. coli* expressing *bla*_IMP__-__70_ showed higher MICs for doripenem and meropenem, but the same MICs for imipenem and panipenem, than *E. coli* expressing *bla*_IMP__-__1_. *E. coli* expressing *bla*_IMP__-__10_ showed a significantly higher MIC for doripenem and an increased MIC for meropenem. Assessment of the carbepenemase activities of recombinant IMP-1, IMP-10, IMP-1(Phe87Val) and IMP-70 showed that IMP-10 had greater hydrolytic activities than IMP-1 against meropenem, with the *k*_cat_/*K_m_* values of IMP-70 and IMP-10 being 2.3- and 3.4-fold higher, respectively, than those of IMP-1 (Table 5). In contrast IMP-70 and IMP-1 showed similar carbapenemase activities against doripenem, imipenem and panipenem, and IMP-1(Phe87Val) showed similar or reduced carbapenemase activities against all carbapenems tested.

**Figure 2:**
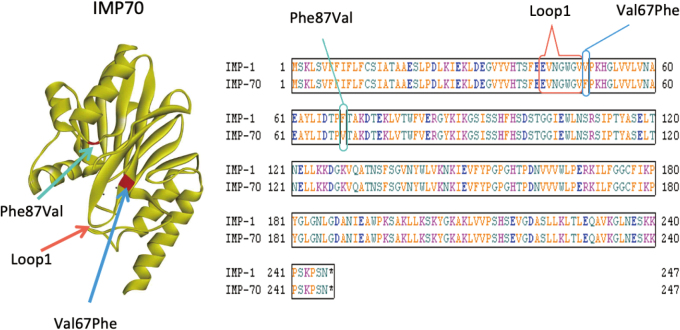
3D structure of IMP-70 MBL and amino acid sequences of IMP-1 and IMP-70 MBLs

These results suggest that, in IMP-70, the Val67Phe amino acid substitution, but not the Phe87Val substitution, is important for the significantly increased carbapenemase activity against meropenem. The Val67 in IMP-1 is located at the end of “loop1”, close to the active site consisting of amino acids residues 60 to 66 (Figure 2)^19)^. Loop1 is a major determinant for the tight binding of substrates in the active site^19)^. A Val67Phe amino acid substitution in IMP-43, a variant of IMP-7, has been reported to increase catalytic activities against imipenem and meropenem^20)^. Amino acid substitutions at residue 67 in IMP-1 MBLs affect their hydrolytic activity against β-lactams^21)^. Residue 67 was reported to be important for substrate binding in VIM-type MBLs^22)^. Residue 87 plays a crucial role in the stability of VIM-2^23)^. IMP-44, a variant of IMP-11 with two substitutions (Val67Phe and Phe87Ser), had more efficient catalytic activities against carbapenems than those of IMP-11^24)^. These results suggest that co-occurrence of two amino acid substitutions at these two positions increase the enzymatic activities of IMP-44, whereas the Phe87Val substitution did not affect the enzymatic activities of IMP-70. The substitution of Phe87 by a polar amino acid such as Ser, but not by a hydrophobic amino acid such as Val, may affect enzymatic activities.

**Table 3:** Drug susceptibility profiles of *Providencia* species clinical isolates

Antibiotic	MIC(μg/ml)					
	*P. rettgeri*					*P. stuartii*
	BML2496	BML2531	BML2526	BML2576		BML2537
Imipenem	16	32	512	512		>512
Meropenem	64	32	512	512		512

**Table 4:** Drug susceptibility profiles of *E. coli* expressing IMP-1, IMP-10, a variant of IMP-1 with an amino acid substitution (F87V) and IMP-70^a^

	MIC(μg/ml)				
antibiotic(s)	*E.coli* DH5α (pHSG398）	*E.coli* DH5α (pHSG398/IMP-1)	*E.coli* DH5α (pHSG398/IMP-10)^b^	*E.coli* DH5α (pHSG398/IMP-1(F87V))	*E.coli* DH5α (pHSG398/IMP-70)
Doripenem	≤0.06	2	8	1	4
Imipenem	0.125	1	1	1	1
Meropenem	≤0.06	4	8	2	8
Panipenem	0.125	2	2	1	2

^a^ Iwata S, Tada T, Hishinuma T, et al: Antimicrob Agents Chemother, 2020; 64.^18)^ ^b^ IMP-10 and IMP-1(V67F) amino acid arrays are the same

**Table 5:** Kinetic parameters of β-lactamases IMP-1, IMP-10, a variant of IMP-1 with an amino acid substitution (F87V) and IMP-70 with substrates^a^

	*kcat/Km* (μM-1・s-1)^b^			
Substrate	IMP-1	IMP-10	IMP-1(F87V)	IMP-70
Doripenem	0.13	0.82	0.091	0.18
Imipenem	0.23	0.24	0.17	0.24
Meropenem	0.15	0.51	0.17	0.35
Panipenem	0.40	0.35	0.24	0.23

^a^ Iwata S, Tada T, Hishinuma T, et al: Antimicrob Agents Chemother, 2020; 64.^18)^ ^b^ *Km* and *k*cat were calculated as means ± SD from three independent experiments.

## Biological significance of two copies of *bla*_IMP__-__1_ in tandem

**Figure 3:**
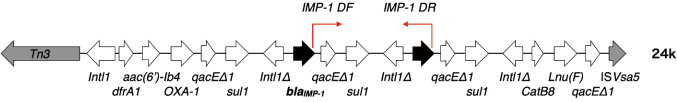
Genomic environments of *bla*_IMP__-1_ and *bla*_IMP-70_ in clinical isolates of *P. rettgeri* and *P. stuartii*. Genes are represented as arrows, which indicate their transcription orientations and relative lengths. MBL genes, *tnp* genes, and truncated genes are shown as black arrows, gray arrows, and Δ, respectively. Label *orf1* represents a gene encoding a hypothetical protein, and *orf2* represents a gene encoding an ATP-binding protein. This figure is a modified version of FIG 1 in reference 18. (Iwata S, Tada T, Hishinuma T, et al: Emergence of Carbapenem-Resistant *Providencia rettgeri* and. Antimicrob Agents Chemother, 2020; 64.)

**Figure 4:**
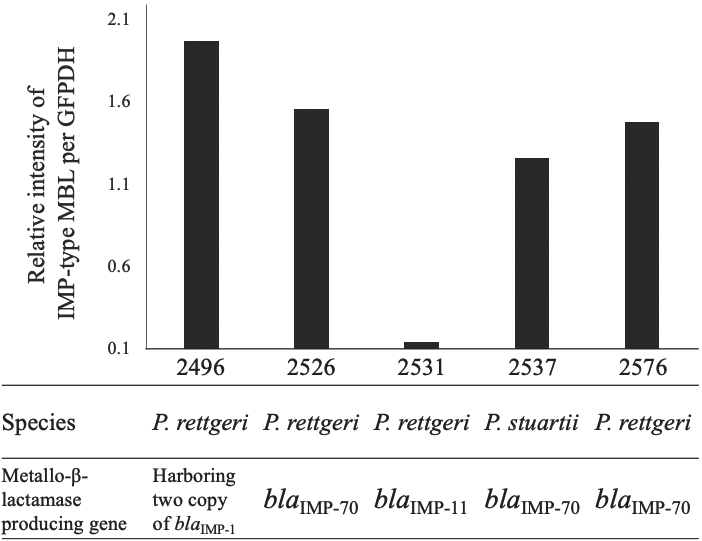
IMP-type MBL production in carbapenem-resistant clinical isolates of *P. rettgeri* and *P. stuartii.* Four clinical isolates of *P. rettgeri* (2496, 2526, 2531 and 2576) and one of *P. stuartii* (2537) were solubilized and subjected to western blot analysis using monoclonal antibodies against IMP-type MBL and GAPDH. The relative intensity of IMP-type MBL bands to GAPDH bands was calculated. This figure is a modified version of FIG 2 in reference 18. (Iwata S, Tada T, Hishinuma T, et al: Emergence of Carbapenem-Resistant *Providencia rettgeri* and. Antimicrob Agents Chemother, 2020; 64.)

One of the *P. rettgeri* isolates was found to harbor two copies of *bla*_IMP__-__1_, in tandem on the chromosome, consisting of a repeat of the genetic structure *int1*Δ-*bla*_IMP__-__70_-*qacE*Δ1-*sul1* (Figure 3). To confirm the presence of the two copies of *bla*_IMP__-__1_, sequences were amplified by PCR using a primer set targeting the two copies. Amplification resulted in a 3.5-kbp PCR product as expected based on the whole-genome sequence, indicating that this isolate of *P. rettgeri* harbored two tandem copies of *bla*_IMP__-__1_ on the chromosome. Western blotting analysis revealed that all five isolates tested produced IMP- type MBLs (Figure 4). Of these five isolates, the *P. rettgeri* isolates harboring two copies of *bla*_IMP__-__1_ produced the largest quantities of IMP-type MBL (Figure 4), indicating these two copies of *bla*_IMP__-__1_ produce high amounts of IMP-1 MBL.

## Conclusions

The genus *Providencia*, belonging to the family *Morganellaceae*, consists of 10 species. Of these, three species, *P. alcalifaciens*, *P. rettgeri* and *P. stuartii*, are clinically important. *P. alcalifaciens* causes diarrhea by invading the intestinal mucosa, whereas *P. stuartii* and *P. rettgeri* have been found to cause hospital acquired urinary tract infections, as well as pneumonia, meningitis, endocarditis, wound infections, bloodstream infections, and travelers’ diarrhea. Clinical isolates of carbapenem- resistant *P. rettgeri* producing IMP-1 MBL were first identified during laboratory-based surveillance in 2000 in Japan. To date, there have been 16 reports of carbapenem-resistant *P. rettgeri*, eight of carbapenem-resistant *P. stuartii* and one of carbapenem-resistant *P. vermicola*, with most of these being clinical isolates.

We recently obtained four *P. rettgeri* isolates and one *P. stuartii* isolate from urine samples of five patients. All five isolates were highly resistant to carbapenems. Three isolates harbored *bla*_IMP__-__70_, encoding a variant of IMP-1 MBL with two amino acid substitutions, and one each harbored *bla*_IMP__-__1_ and *bla*_IMP__-__11_. Molecular analyses of these isolates strongly suggest that *Providencia* species become more highly resistant to carbapenems by acquisition of two copies of *bla*_IMP__-__1_ or by mutations in *bla*_IMP_ genes that result in amino acid substitutions, such as *bla*_IMP__-__70_.

## Funding

This study was supported by grants from Japan Society for the Promotion of Science (grants 18K07120 and 19K16652) and Research Program on Emerging and Re-emerging Infectious Diseases from Japan Agency for Medical Research and Development (grant number 22fk018604h702). S.I. was supported by the Training Program for Medical Students in Basic Research, Ministry of Education, Culture, Sports, Science and Technology (MEXT), Japan (student number 2117016).

## Author's contributions

SI collected and reviewed the presented data from previously published articles in medical journals, and drafted the manuscript. TT reviewed the data on drug-resistant genes. SO analyzed the data on biochemical experiments. TH supervised the data on drug-susceptibility profiles. MT constructed the phylogenetic tree and drafted the section of bacterial taxonomy. TT supervised this study.

## Conflicts of interest statement

We have the following interests. Drs. Miho Ogawa and Masahiro Shimojima is employed by BMI Inc. There are no patents, products in development or marketed products to declare.
